# Effect of Opium Addiction on Postoperative Arrhythmia Among Patients Undergoing CABG operation on Cardiopulmonary Bypass

**DOI:** 10.21470/1678-9741-2018-0392

**Published:** 2019

**Authors:** Majid Basirat, Mohsen Ziyaeifard, Farhad Gorjipour, Mohammad Javad Mehrabanian, Seyed Hassan Attarzadeh, Majid Nekoofard, Meysam Mortazian

**Affiliations:** 1 Sary Branch, Islamic Azad University, Sary, Iran.; 2 Rajaie Cardiovascular, Medical and Research Center, Iran University of Medical Sciences, Tehran, Iran.; 3 Tehran Heart Center, Tehran University of Medical Sciences, Tehran, Iran.; 4 AJA University of Medical Sciences, Tehran, Iran.

**Keywords:** Coronary Artery Bypass, Arrhythmias, Cardiac, Opium Dependence, Central Venous Pressure, Epinephrine, Postoperative Period

## Abstract

**Objectives:**

Postoperative arrhythmia is an important complication of coronary artery bypass grafting (CABG) surgeries among patients. It seems that opioid usage is implicated in the pathogenesis of this condition due to its impacts on different organ systems, such as the autonomic nervous system. The present study was performed to investigate the effect of opium use on postoperative arrhythmia in patients undergoing CABG surgery.

**Methods:**

Study participants were selected via convenience sampling from patients undergoing CABG surgery in a referral hospital. Study variables, including use of inotropic drugs, vital signs monitoring parameters and postoperative arrhythmia were observed and recorded at baseline and at follow-up time after surgery.

**Results:**

Sixty-five (14.8%) patients had postoperative arrhythmia, and 104 participants were addicted. Prevalence of postoperative arrhythmia was the same among addict and non-addict patients. According to the regression analysis model, only serum level of epinephrine in operating room, heart rate and central venous pressure at baseline and 48 hours after operation are known as independent predictors of postoperative arrhythmia among study population.

**Conclusion:**

This study showed that although opium addiction increased postoperative arrhythmia among patients undergoing CABG surgery, this difference was not significant, and this association is probably mediated by other study variables.

**Table:** 

Abbreviations, acronyms & symbols	
CAD	= Coronary artery disease
CABG	= Coronary artery bypass grafting
CPB	= Cardiopulmonary bypass
CVD	= Cardiovascular diseases
ICU	= Intensive care unit
OR	= Operating room
WHO	= World Health Organization

## INTRODUCTION

Prevalence of cardiovascular diseases (CVD) has increased in recent years. Fifty percent of deaths in developed countries and 25% of deaths in developing countries are related to CVD^[1]^. According to the World Health Organization’s (WHO) reports, CVD causes one third of all annual deaths around the world^[2]^. Coronary artery bypass grafting (CABG) surgery is performed in CVD patients to improve their outcomes and to decrease incidence of mortality. Postoperative arrhythmia is one of the CABG complications, and different studies have been performed to the management of this complication among patients undergoing cardiopulmonary surgery^[3,4]^.

Drug addiction is known as one of the major social health problems in the world. Opium dependence is highly prevalent among Iranian drug abusers^[5,6]^. As an example, according to a large clinical survey, around 5.2% of Iranian patients with CVD were opium-dependent^[7]^. It seems that there is a misconception among Iranian population that opium usage might decrease adverse impacts of hypertension, diabetes mellitus and CVD^[8]^.

Although most of the effects of opium are focused on the central and autonomic nervous system and the intestinal tract, opium usage may have impacts on some other organ systems, such as respiratory and cardiovascular systems^[9]^. Effects of opium usage on the cardiovascular system are known to be mediated by some endogenous ligands as opioid peptides^[10]^. Opioid peptides interact with most of the pathophysiological cardiac outcomes, such as tachycardia/bradycardia, hypotension/hypertension, and vasodilation. According to our search in the literature, there were few papers available regarding impacts of opioid usage on postoperative outcomes such as arrhythmia. We assumed that opioid usage, due to its impacts on different organs, such as autonomic nervous system, is potentially a risk factor in postoperative arrhythmia among patients undergoing cardiac surgeries. The present study was performed to evaluate the effect of opium consumption on postoperative arrhythmia among patients undergoing CABG.

## METHODS

### Study Design and Patient Selection

Present clinical research was performed on patients undergoing CABG surgery in Rajaie Cardiovascular Medical and Research Center (Tehran, Iran). Study population was selected via convenience sampling from patients who were referred to heart surgery clinic for CVD management and were candidates for elective CABG surgery. Study inclusion criteria were elective CABG surgery, age between 18 and 85 years, male or female. Patients with prior history of cardiopulmonary bypass, known cardiac rhythm disturbances, use of pacemakers or advanced heart failure were excluded from the study. Study protocol was designed according to the Declaration of Helsinki and was approved by the Ethics Committee of Iran University of Medical Sciences. Informed consent was obtained from all study participants before starting the trial.

### Data Collection

Data related to patient demographics and history were extracted and recorded in standard checklists. Then a checklist for each patient was used for data collection, and data were taken and recorded by a nursing professional. Use of inotropic drugs such as epinephrine, norepinephrine, dopamine, milrinone and dobutamine before, during and after surgery was recorded. Vital signs, including systolic and diastolic blood pressure, heart rate and central venous pressure, were measured at baseline, after surgery, in the ICU admission, and 6, 24 and 48 hours after surgery. Heart rhythm of the patients was assessed until 72 hours after operation and occurrence of dysrhythmia was recorded, and their types were assessed as dependent variables.

Patients were grouped according to the status of opium addiction or the status of arrhythmias after operation.

### Statistical Analysis

Data were analyzed using SPSS V20 software for statistical analysis. Frequencies of study variables were compared in the two groups with chi-square and *odds ratio*. Quantitative variables were presented as mean ± standard deviation, and qualitative variables were presented as frequencies and frequency percentage. Independent sample Student’s t-test was used for comparing the mean of continuous study variables between the two groups. We used logistic regression model to assess the role of study variables in the incidence of postoperative arrhythmia. We included all variables that had a *P*-value <0.2 in their univariate association with postoperative arrhythmia. All *P*-values <0.05 were assumed as significant results.

## RESULTS

Finally, 438 patients (222 female) met our inclusion criteria and were included in the trial. Mean age, body mass index and preoperative left ventricle ejection fraction were 61.23±9.05 years, 25.26±4.54 kg/m^2^ and 41.91%±9.26%, respectively. Among trial participants, 306 patients (69.7%) had comorbidities including diabetes (47.6%), hypertension (56.9%), thyroid disorders (6.6%), and renal (9.3%) and neurological lesions (4.6%). The mean surgery time was 265.02±76.61 minutes and the mean cardiopulmonary bypass (CPB) time was 86.57±32.85. Among comorbidities, only diabetes (*P*=0.008) had a significant association. Hypertension (*P*=0.34), thyroid (*P*=0.16), neurological (*P*=0.67) and renal disorders (*P*=0.08) had no significant association with the incidence of postoperative arrhythmia.

In total, 104 participants (23.8%) were addicted. Mean CPB time (89.16±3.02 *vs*. 85.89±31.95; *P*=0.59) and mean cross-clamping time (49.15±24.01 *vs*. 45.83±20.40; *P*=0.38) among addict patients were not significantly lower than other patients. The mean surgery time among addict patients was significantly lower than other patients (248.85±80.02 *vs*. 269.60±74.94; *P*=0.03). Among inotropic drugs, only mean of epinephrine (*P*=0.02) and dopamine (*P*=0.01) in operating room (OR) were significantly different among patients with postoperative arrhythmia and other patients. When comparing the use of inotropic drugs among addicts and other patients, except epinephrine in OR and ICU, other drugs did not present significant changes (Tables 1 and 2).

**Table 1:** Inotropic and anticoagulant drug usage between study population with and without postoperative arrhythmia.

	POA (+)	POA (-)	*P*-value^*^
Epinephrine in OR	0.77±0.42	0.84±0.37	0.02
Epinephrine in ICU	0.74±0.44	0.83±0.37	0.11
Norepinephrine in OR	1±0.001	0.98±0.11	0.41
Norepinephrine in ICU	1±0.001	0.97±0.11	0.41
Dopamine in OR	0.94±0.24	0.98±0.12	0.01
Dopamine in ICU	0.96±0.17	0.98±0.14	0.53
Milrinone in OR	0.97±0.17	0.99±0.09	0.12
Milrinone in ICU	1.01±0.02	0.99±0.09	0.46
Dobutamine in OR	0.97±0.17	0.98±0.11	0.21
Dobutamine in ICU	1.0±0.001	0.99±0.08	0.55

* *P*-value was calculated according to independent Student's t-test

ICU=intensive care unit; OR=operating room; POA=postoperative arrhythmia

**Table 2:** Inotropic and anticoagulant drug usage between study population.

	Addict patients	Non-addict patients	*P*-value^*^
Epinephrine in OR	0.92±0.27	0.78±0.41	0.004
Epinephrine in ICU	1±0.001	0.98±0.11	0.001
Norepinephrine in OR	1±0.001	0.11±0.98	0.263
Norepinephrine in ICU	1±0.001	0.97±0.11	0.263
Dopamine in OR	1±0.001	0.97±0.16	0.091
Dopamine in ICU	1±0.001	0.96±0.16	0.091
Milrinone in OR	0.99±0.09	0.98±0.1	0.084
Milrinone in ICU	0.98±0.15	0.34±0.91	0.97
Dobutamine in OR	1.0±0.001	0.98±0.13	0.16
Dobutamine in ICU	1.0±0.001	0.99±0.08	0.42

* *P*-value was calculated according to independent Student's t-test.

ICU=intensive care unit; OR=operating room

To see if there is a difference between addict and non-addict patients, they were divided into two groups and compared for their demographic information. No significant differences were found between the two groups according to their demographics (Table 3).

**Table 3:** Comparison of demographic variables between addict and non-addict study population.

	Addict patients (n=104)	Non-addict patients (n=332)	*P*-value^*^
Age	60.36±8.69	61.49±9.17	0.27
BMI	25.94±4.72	26.38±4.49	0.41
Sex (male)	53 (24.7%)	162 (75.3%)	0.70
Diabetes	52 (25.1%)	155 (74.9%)	0.56
Hypertension	62 (24.9%)	187 (75.1%)	0.53
Thyroid disorders	3 (10.7%)	25 (89.3%)	0.09
Neurological lesions	4 (20%)	16 (80%)	0.80
Renal lesions	12 (29.3%)	29 (70.7%)	0.40

* *P*-value was calculated according to independent Student's t-test and chi-square test.

**Fig. 1:**
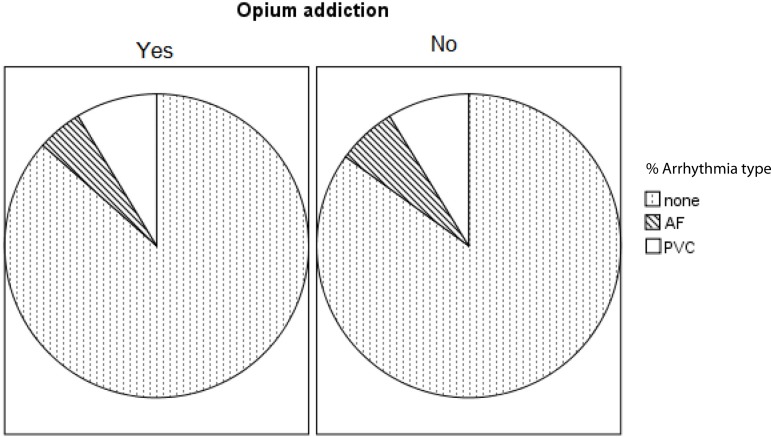
Frequency percentage of patients with each type of arrhythmia in patients with and without drug addiction. There were no statistically significant differences between the two groups in the arrhythmia type. AF=atrial fibrillation; PVC=premature ventricular contractions

In this study, 65 (14.8%) patients had postoperative arrhythmia. Prevalence of postoperative arrhythmia was similar among addict and non-addict patients (15, 14.4% *vs*. 50, 15.1%; OR: 0.96 95% CI: 0.56-1.63; *P*=0.87). When accounting for the type of arrhythmia, there were also no significant differences between patients with and without history of opium addiction. Mean age (60.01±8.39 *vs*. 61.29±9.06; *P*=0.74) and mean body mass index (26.18±3.46 *vs*. 26.26±4.56; *P*=0.96) were similar between patients with and without postoperative arrhythmia. Premature ventricular contractions are the most prevalent arrhythmias in both groups, followed by atrial fibrillation. However, there was no significant difference between the two groups in terms of type of arrhythmia (Figure 1). Systolic and diastolic blood pressure and heart rate at baseline, systolic blood pressure at 6 and 48 hours after surgery and heart rate in the ICU admission were significantly different among patients with postoperative arrhythmia and other patients (Table 4).

**Table 4:** Comparing blood pressure and heart rate among study population in different study points between patients with and without postoperative arrhythmia.

	POA (+)	POA (-)	*P*-value
SBP at baseline	135.36±22.54	123.32±19.63	<0.001
DBP at baseline	75.18±11.78	71.14±12.33	0.02
SBP after surgery	120.74±16.24	116.64±18.30	0.12
DBP after surgery	70.64±18.30	69.02±11.93	0.34
SBP at ICU admission	119.03±23.86	116.43±21.09	0.37
DBP at ICU admission	68.81±11.69	66.52±13.49	0.21
SBP 6 hours later	120.59±17.01	114.19±14.04	0.001
DBP 6 hours later	69.29±11.39	66.05±12.73	0.06
SBP 24 hours later	118.94±17.69	115.58±14.73	0.09
DBP 24 hours later	68.62±16.76	66.40±11.84	0.20
SBP 48 hours later	121.51±20.55	117.28±13.56	0.04
DBP 48 hours later	70.01±11.34	66.95±12.62	0.07
HR at baseline	75.65±11.28	79.20±11.12	0.03
HR after surgery	82.65±11.28	83.97±11.53	0.44
HR at ICU admission	91.66±16.64	86.10±13.45	0.01
HR 6 hours later	87.17±16.64	84.96±10.83	0.17
HR 24 hours later	86.51±16.50	83.23±11.21	0.09
HR 48 hours later	83.38±14.28	83.18±10.08	0.89
CVP at baseline	13.73±15.45	11.35±9.96	0.13
CVP after surgery	10.42±2.65	9.72±2.86	0.08
CVP at ICU admission	10.62±4.21	10.38±3.88	0.66
CVP 6 hours after surgery	11.63±3.44	12.61±8.36	0.35
CVP 24 hours after surgery	14.32±5.82	14.02±12.43	0.85
CVP 48 hours after surgery	14.01±3.78	13.01±3.46	0.06

CVP=central venous pressure; DBP=diastolic blood pressure; HR=heart rate; ICU=intensive care unit; POA=postoperative arrythmia; SBP=systolic blood pressure

### Logistic Regression Analysis Findings

After the inclusion of selected variables in the logistic regression model, only serum levels of epinephrine in OR, heart rate and central venous pressure at baseline and 48 hours after surgery remained in the regression model and were known as independent predictors of postoperative arrhythmia among the study population (Table 5).

**Table 5:** Regression analysis for determination of independent predictors of postoperative arrhythmia among study participants.

Included variables	B	*P*-value	Exp (B)	95% CI for Exp (B)	
				Lower	Upper
Age	-0.04	0.19	0.96	0.91	1.02
Surgery time	-0.003	0.45	0.99	0.99	1.005
Diabetes (1)	0.37	0.58	1.45	0.39	5.41
Thyroid (1)	0.46	0.64	1.59	0.22	11.52
Renal (1)	-1.98	0.06	0.14	0.017	1.08
Epinephrine in OR	-1.67	0.091	0.19	0.027	1.30
Epinephrine in ICU	2.5	0.01	12.55	1.83	86.37
Dopamine in OR	0.89	0.59	2.44	0.095	62.54
Milrinone in OR	24.68	0.99	5.24	0.001	0.99
SBP at baseline	-0.007	0.68	0.99	0.96	1.03
DBP at baseline	-0.054	0.15	0.95	0.88	1.02
Postoperative SBP	-0.022	0.25	0.98	0.94	1.02
SBP 6 hours after surgery	0.004	0.85	1.004	0.96	1.04
DBP 6 hours after surgery	0.009	0.74	1.009	0.96	1.06
SBP 48 hours after surgery	-0.013	0.52	0.99	0.95	1.03
DBP 48 hours after surgery	-0.026	0.29	0.98	0.93	1.02
HR at baseline	0.058	0.04	1.06	1.002	1.122
HR at ICU	-0.019	0.37	0.98	0.94	1.02
HR 6 hours after surgery	0.040	0.24	1.04	0.97	1.11
HR 24 hours after surgery	-0.93	0.004	0.91	0.86	0.97
CVP baseline	-0.050	0.03	0.95	0.91	0.99
Postoperative CVP	-0.084	0.47	0.91	0.73	1.15
CVP 48 hours after surgery	-0.175	0.04	0.83	0.71	0.99
Constant	-5.480	1	0.0040	-	-

CVP=central venous pressure; DBP=diastolic blood pressure; HR=heart rate; ICU=intensive care unit; OR=operating room; SBP=systolic blood pressure

## DISCUSSION

In this study, 65 (14.8%) patients presented postoperative arrhythmia and 104 participants were addicted. Prevalence of postoperative arrhythmia was similar among addict and non-addict patients. According to the regression analysis model, only serum levels of epinephrine in OR, heart rate and central venous pressure level at baseline and 48 hours after surgery were demonstrated to be independent predictors of postoperative arrhythmia among study population.

There were several studies which reported opium addiction among different populations^[10,11]^. As an example, prevalence of smoking was 38.5% among Iranian patients undergoing surgery^[12]^. In one study, the prevalence of smoking among CABG patients was 37.5%^[13]^. There are different studies with conflicting findings on the association of smoking or opium addiction with CABG postoperative outcomes. Many similar studies reported that higher morbidity and mortality rates were found among smokers than nonsmokers after cardiac surgery^[12,14]^. Although most studies reported that the risk of CAD increased with opium usage, in one study in 2004, CAD severity was lower among methadone or opiate users in comparison with other patients. On the other hand, researchers believed that long-time opium exposure can decline the severity of CAD and fatal outcome^[15]^.

Our study findings showed similar postoperative arrhythmia among addict and non-addict participant patients. We discussed cautiously about findings of similar studies due to lack of some essential information, such as history of smoking, lipid profile, and lifestyle among addict patients. In other studies, opium usage has been shown an independent predictor of coronary artery disorders among patients and has even correlated with the number of diseased vessels^[16]^. In another study, investigators found that opium consumption was a major risk factor for acute myocardial infarction^[17]^. When comparing the operating time between two groups of addict and non-addict patients, a significantly shorter surgery time was observed in addicts. This difference may be due to chance or due to the patients’ tolerance to analgesics and opioid anesthetics or other analogs. However, regression analysis revealed no relationship between surgery time and the occurrence of arrhythmias postoperatively in the present study.

Different studies in the literature compare outcomes of CABG surgery among opium users and other patients. They reported that postoperative complications and duration of hospital stay were similar between the two groups, but opium users were more likely than other patients to be readmitted in the hospital due to cardiac complications during the six-month follow-up^[18]^. Some methodological bias is found among these clinical studies. Most of the studies had case-control or cross-sectional design. Although a higher prevalence of opium consumption observed among patients with coronary artery disease than in controls, a causal interpretation is not warranted due to the gap between occurrence and exposure times.

Another significant observation was the higher consumption of inotropic agents in patients without postoperative arrhythmia. This observation can be described as a consequence of hemodynamic instability and lower preoperative blood pressure in these patients. Consumption of these agents was also higher in addicts in the OR and in the ICU. This is probably due to the higher doses of opioids required for analgesia in these patients. Moreover, some of them probably abuse more of these opioids in the hospital without the physician’s order. Therefore, these patients will present lower blood pressure and will need epinephrine administration to stabilize the hemodynamics.

However, interpretation of the findings of the present study must be very conservative and inferences about the relationship between these variables are limited to an association rather than to causality. Cohort or longitudinal study for approving causal association of opium addiction and postoperative outcomes among patients undergoing cardiac surgery is warranted.

## CONCLUSION

Our study demonstrated that despite the increase in the rate of postoperative arrhythmia among opium-addicted patients undergoing CABG surgery, this difference was not significant, and this association is mediated by other study variables.

**Table:** 

Author's roles & responsibilities	
MB	Substantial contributions to the conception or design of the work; or the acquisition, analysis, or interpretation of data for the work; drafting the work or revising it critically for important intellectual content; agreement to be accountable for all aspects of the work in ensuring that questions related to the accuracy or integrity of any part of the work are appropriately investigated and resolved; final approval of the version to be published
MZ	Substantial contributions to the conception or design of the work; or the acquisition, analysis, or interpretation of data for the work; drafting the work or revising it critically for important intellectual content; agreement to be accountable for all aspects of the work in ensuring that questions related to the accuracy or integrity of any part of the work are appropriately investigated and resolved; final approval of the version to be published
FG	Substantial contributions to the conception or design of the work; or the acquisition, analysis, or interpretation of data for the work; drafting the work or revising it critically for important intellectual content; agreement to be accountable for all aspects of the work in ensuring that questions related to the accuracy or integrity of any part of the work are appropriately investigated and resolved; final approval of the version to be published
MJM	Substantial contributions to the conception or design of the work; or the acquisition, analysis, or interpretation of data for the work; drafting the work or revising it critically for important intellectual content; agreement to be accountable for all aspects of the work in ensuring that questions related to the accuracy or integrity of any part of the work are appropriately investigated and resolved; final approval of the version to be published
SHA	Substantial contributions to the conception or design of the work; or the acquisition, analysis, or interpretation of data for the work; drafting the work or revising it critically for important intellectual content; agreement to be accountable for all aspects of the work in ensuring that questions related to the accuracy or integrity of any part of the work are appropriately investigated and resolved; final approval of the version to be published
MN	Substantial contributions to the conception or design of the work; drafting the work or revising it critically for important intellectual content; agreement to be accountable for all aspects of the work in ensuring that questions related to the accuracy or integrity of any part of the work are appropriately investigated and resolved; final approval of the version to be published
MM	Substantial contributions to the conception or design of the work; or the acquisition, analysis, or interpretation of data for the work; drafting the work or revising it critically for important intellectual content; agreement to be accountable for all aspects of the work in ensuring that questions related to the accuracy or integrity of any part of the work are appropriately investigated and resolved; final approval of the version to be published
